# Secondary Impact of Social Media via Text Message Screening for Type 2 Diabetes Risk in Kuwait: Survey Study

**DOI:** 10.2196/20532

**Published:** 2020-11-12

**Authors:** Naeema Alqabandi, Ebaa Al-Ozairi, Adel Ahmed, Edgar L Ross, Robert N Jamison

**Affiliations:** 1 Dasman Diabetes Institute Kuwait City Kuwait; 2 Pain Management Center Brigham and Women's Hospital Harvard Medical School Chestnut Hill, MA United States

**Keywords:** SMS, Short text message interventions, mHealth, smartphone, Type 2 diabetes mellitus, prevention

## Abstract

**Background:**

Type 2 diabetes mellitus (T2DM) is an international problem of alarming epidemic proportions. T2DM can develop due to multiple factors, and it usually begins with prediabetes. Fortunately, this disease can be prevented by following a healthy lifestyle. However, many health care systems fail to properly educate the public on disease prevention and to offer support in embracing behavioral interventions to prevent diabetes. SMS messaging has been combined with cost-effective ways to reach out to the population at risk for medical comorbidities. To our knowledge, the use of nationwide SMS messaging in the Middle East as a screening tool to identify individuals who might be at risk of developing T2DM has not been reported in the literature.

**Objective:**

The primary aim of this study was to assess the feasibility of conducting a series of SMS messaging campaigns directed at random smartphone users in Kuwait for the detection and prevention of T2DM. It was predicted that 1% of those receiving the text message would find it relevant and participate in the study. The secondary aim of this study was to assess the incidence of participation of those who were forwarded the initial text message by family members and friends.

**Methods:**

In this study, 5 separate text message screening campaigns were launched inviting recipients to answer 6 questions to determine the risk of developing T2DM. If subjects agreed to participate, a link to the prediabetes screening test devised by the Centers for Disease Control and Prevention was automatically transmitted to their mobile devices. Those identified as high risk were invited to participate in a diabetes prevention program.

**Results:**

A total of 180,000 SMSs were sent to approximately 6% of the adult population in Kuwait. Of these, 0.14% (260/180,000) of the individuals who received the SMS agreed to participate, of whom 58.8% (153/260) completed the screening. Surprisingly, additional surveys were completed by 367 individuals who were invited via circulated SMS messages forwarded by family members and friends. Altogether, 23.3% (121/520) qualified and agreed to participate in a diabetes prevention program. The majority of those who chose to participate in the prevention program were overweight, aged 45-65 years, and reported being less physically active than those who chose not to participate (*χ*^2^_2_=42.1, *P*<.001).

**Conclusions:**

Although health care screening via text messaging was found to have limited effectiveness by itself, it exhibited increased reach through shared second-party social media messaging. Despite the fact a subpopulation at possible risk of developing T2DM could be reached via text messaging, most responders were informed about the screening campaign by family and friends. Future research should be designed to tap into the benefits of social media use in health risk campaigns.

## Introduction

Type 2 diabetes mellitus (T2DM) is an international problem of alarming epidemic proportions [1]. The causes of T2DM can be multifactorial, with prediabetes tending to be the predominant one [2]. Prediabetes is an asymptomatic phase in which blood glucose levels are higher than normal but not high enough to be diagnosed with T2DM, characterized by insulin resistance, or both. This phase is associated with either one or both kinds of glucose levels: fasting glucose and impaired glucose tolerance [3]. Environmental and genetic factors as well as a sedentary lifestyle and unhealthy eating habits are known to play key roles. Diabetes mellitus can cause serious chronic medical comorbidities, including heart and blood vessel disease, blindness, neuropathy, limb amputation, and kidney failure [4]. Fortunately, this disease can be prevented by following a healthy lifestyle [5,6]. However, many health care systems fail to properly educate the public on disease prevention and to offer support in embracing behavioral interventions to prevent diabetes.

In 2016, Kuwait was known to have one of the highest percentages of people who lived a sedentary lifestyle, estimated to be 67% of the total population [7,8]. This is due in part to a hot climate (the average daily temperature reaches 45 °C or 113 °F in summer [9]), an overreliance on motor transportation, readily affordable domestic labor, and the advent of technology that encourages less daily physical activity. In addition, an abundance of food supplied on all social occasions and a focus on eating indicative of growing financial affluence contribute to higher rates of obesity [10-13].

SMS messaging has proved to be a cost-effective way to reach out to the population at risk for medical comorbidities, including asthma, hypertension, HIV, and diabetes [14-18]. SMS messaging is rapidly becoming an important communication vehicle worldwide for reaching the general population [19]. SMS messaging is a feasible, accessible, and cost- and time-effective method that ensures instant transmission to the recipient, which can be confirmed through 2-way messaging [20,21]. Mobile SMS, a segment of the mobile health strategy, can serve as a mediator between health care providers and the public [19-22]. Leading health organizations have recommended the use of text messaging in health care settings [23,24]. SMS effectiveness has been examined in previous studies focusing on 2 main domains: behavior change interventions and reminders [25,26]. Unfortunately, according to Gallup polling, SMS text messaging has a lower response rate than telephone surveys, and the percent of response can be as low as 1-2% if the individuals who were sent a general message had never been contacted before [27].

To our knowledge, the use of nationwide SMS messaging in the Middle East as a screening tool to identify individuals who might be at risk of developing T2DM has not been reported in the literature. The impact of circulating short text messages as part of health campaigns to reach those who may have prediabetes, asymptomatic diabetes, or are at high risk for diabetes is unknown. The primary aim of this study was to assess the feasibility of conducting a series of SMSs campaigns among random smartphone users in Kuwait for the detection and prevention of T2DM. It was predicted that 1% of those receiving the text message would find it relevant and participate in the study. The secondary aim of this study was to assess the incidence of participation of those who were forwarded the initial text message by family members and friends.

## Methods

### Study Design

This pilot study was designed to evaluate the impact of separate SMS messaging health care campaigns directed at owners of smartphones in 6 main governorates in Kuwait. This approach was the initial step of a larger study aimed to help identify and assist persons at risk of developing T2DM. The screening program offered instructions for obtaining confirmatory diagnostic blood testing once it was determined that an individual was at high risk for T2DM. We decided to send text messages to the general population in order to reach potential participants because of the easy accessibility of phone numbers through the national telephone service. Ethics approval for the study was obtained from the Dasman Diabetes Institute’s local Research Ethical Committee and the Harvard Medical School Institutional Review Board.

### Study Participants

This study was targeted at adults aged 21 years and older, residing in Kuwait, and owning a compatible smartphone (either iPhone or Android). Between October 2017 and December 2018, the telephone company sent an SMS message to 30,000 unique individuals in each of Kuwait’s 6 main governorates on 5 separate occasions, thus totaling to 180,000 messages. The 5 separate SMS campaigns were conducted in October 2017, February 2018, April/May 2018, September 2018, and December 2018. The following SMS message was sent in Arabic and English: “Are you interested in knowing the risks of developing diabetes? Your data will be used for research purposes. If interested, reply YES to this message.” Those who were interested replied in the affirmative to the SMS message. If the participant agreed to participate in the study, a link to an online Centers for Disease Control and Prevention (CDC) questionnaire [28], which is a validated prediabetes screening test measuring the risk of developing T2DM, was automatically transmitted to their mobile devices. The questionnaire is a simple self-assessment that includes the following questions in both English and Arabic:

1. How tall are you? How much do you weigh? (The replies to these questions established the respondent’s BMI.)

2. Is your BMI greater than 27 kg/m^2^?

3. How old are you?

4. Do you have a mother, father, sister, or brother with diabetes?

5. Are you physically active? (Being physically active was defined as conducting physical activity 20 minutes a day, 3 times per week.)

6. Are you male or female? If female, have you ever given birth to a baby that weighed more than 9 lb (or 4 kg), and have you ever had diabetes while pregnant?

The test was scored based on risk factors of weight (people with higher BMIs have a higher risk of developing T2DM) [29], older age, family history of diabetes, inactivity, and gender (more men than women have undiagnosed diabetes) [28]. Each response was weighted, and the score was summarized. Those scoring 9 or more were classified as being at high risk for developing T2DM.

Participants who decided to take the survey were requested to provide their contact number and asked whether they would agree to be contacted by one of the investigators to discuss their scores. Completed surveys were received and accessed by research investigators through Research Electronic Data Capture (REDCap), a secure, web-based software platform designed to support data collection for research studies. The platform provides (1) an intuitive interface for validated data capture, (2) audit trails for tracking data manipulation and export procedures, (3) automated export procedures for seamless data downloads to common statistical packages, and (4) procedures for data integration and interoperability with external sources [30,31]. Eligible and willing respondents who scored 9 points or more on the CDC questionnaire were contacted and asked to participate in a future diabetes prevention intervention program.

### Statistical Analysis

This study was designed to gather data on the feasibility of identifying persons at risk for developing T2DM through SMS text messaging sent to a random list of individuals in the general population of Kuwait. The primary outcome measure was the number of responders of the SMS campaigns. It was expected that 1% of those receiving the text messages would respond based on the literature for general population surveys among persons who had not been contacted before [27]. Secondary analyses were conducted to examine the demographic and mean score differences of the diabetes screening tests between those who were sent a random text message and those who were forwarded the message by family and friends. It was anticipated that those who were sent the message initially would have lower risk scores and a lower rate of response compared with those who received the message through a family member or friend. Secondary analyses were conducted to help identify demographic differences between the initial responders and those who responded after the SMS was shared by a family member or friend. All variables were assessed using bivariate analyses to establish group differences. This study was designed as a preliminary investigation, and no power calculations were performed. The analyses assumed a 2-tailed test and an α level of .05 to confirm the prediction that no differences would be found between the abovementioned groups. Depending on the nature of the variables, nonparametric (chi-square) and parametric (*t* test) analyses were conducted using Bonferroni correctional analyses for multiple comparisons. We also examined and reported on the qualitative responses of the people who participated in the diabetes screening and diabetes prevention program. The data from this study were analyzed to gather information about the utility of SMS messaging for persons at risk for T2DM.

## Results

**Figure 1:**
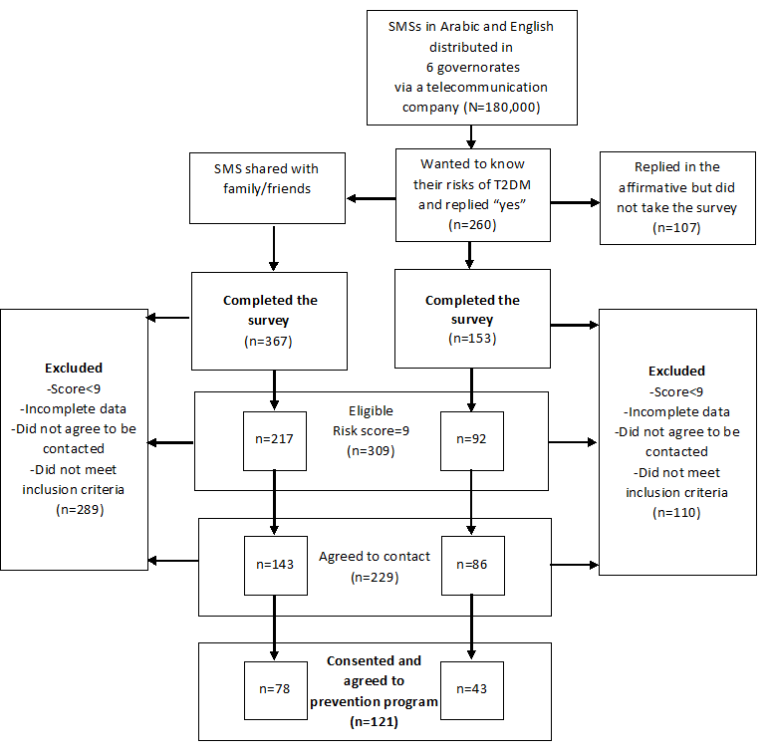
Selection process of eligible participants based on the response to the SMS campaigns and completion of the online survey.

The study schema is presented in Figure 1.

In this study, 5 separate SMS campaigns were conducted, sending out a total of 180,000 text messages, which targeted about 6% of the adult population in Kuwait (Table 1). Of these, 0.14% (260/180,000) of individuals replied “Yes” to the SMS message, indicating that they would like to know more about their risk of developing diabetes. Thus, the overall response rate was 0.14% (260/180,000). Of these individuals, 58.8% (153/260) took the survey. The largest number of responses originated from the fourth campaign (113/36,000, 0.31%). The results showed that 60.1% (92/153) of those who completed the survey were classified as being at high risk for developing T2DM (risk score≥9). 

**Table 1:** Details of respondents for the 5 SMS campaigns originally circulated in 6 governorates in Kuwait between 2017 and 2018.

Type of recruitment		Number of messages sent (N=180,000)	Number who responded (n=260), n (%)	Number who took the survey (n=153), n (%)	Number eligible for prevention study (n=92), n (%)
**SMS campaigns**					
	Campaign 1	36,000	37 (14.2)	13 (5.0)	6 (2.3)
	Campaign 2	36,000	43 (16.5)	22 (8.5)	12 (4.6)
	Campaign 3	36,000	30 (11.5)	15 (5.8)	7 (2.6)
	Campaign 4	36,000	113 (43.5)	76 (29.2)	52 (20.0)
	Campaign 5	36,000	37 (14.2)	27 (10.4)	15 (5.8)

A total of 520 individuals took the survey over the course of the study. Of these, 29.4% (153/520) of individuals completed the survey after receiving the original SMS messages (primary outcome). An additional number of surveys were completed by individuals who were not sent the original SMS message (367/520, 70.6%; secondary outcome). These individuals completed the survey after the text message was circulated by family members, friends, and others. Of all the respondents who completed the survey, 59.4% (309/520) were found to have a risk score≥9. Moreover, 44.0% (229/520) agreed to be contacted, and 23.3% (121/520) of these individuals consented and enrolled in a diabetes prevention study (Figure 1).

Overall, the number of initial responders was very low and below the anticipated 1%. Females agreed to participate in the survey more often than males (349/520, 67.1% vs 171/520, 32.9%; *P*<.001; Table 2). Those between the ages of 21 and 45 years showed the most interest in the study compared to the other age groups (58.3% vs 41.8%). Surprisingly, only 3.3% of those over age 65 showed interest in participating, even though they would be at a higher risk of developing diabetes [32]. Of the subjects who responded, 66% (345/520) reported having parents with T2DM, and 41.6% (209/495) were classified as obese with a reported BMI≥30 [33]. Comparisons were made between those who responded to the initial SMS messaging and those who were informed about the survey through family and friends. Those who were forwarded the message and completed the survey were more often women (258/343, 75.2%; *P*<.001). No other significant differences were found between these two groups. Moreover, 59% (309/520) of the total responders had a risk score≥9, while 36.0% (187/520) scored<9. The remaining 5.2% (27/520) had unknown risk scores due to incomplete and missing data.

**Table 2:** Characteristics of individuals who responded to SMS campaigns (n=520), and differences among those who responded to the initial SMS and completed the survey (n=153) and those who were forwarded the SMS from family and friends and completed the survey (n=343).

Characteristic		Total (n=520)	Initial SMS (n=153)	Family/friends (n=343)	Differences between initial SMS and family/friends	
					Chi-square (*df*)	*t* test (*df*)
Gender (female), n (%)		334 (67.3)	76 (49.7)	258 (75.2)	31.4 (1)^a^	N/A^b^
**Age group (years), n (%)**						
	≥21-<45	292 (58.3)	97 (63.4)	195 (56.9)	2.0 (2)	N/A
	45-65	188 (38.5)	51 (33.3)	137 (39.9)	N/A	N/A
	>65	16 (3.3)	5 (3.3)	11 (3.2)	N/A	N/A
**BMI, mean (SD)**		29.7 (6.0)	30.0 (6.0)	29.6 (6.0)	N/A	0.7 (493)
	Underweight, n (%)	2 (0.4)	1 (0.7)	1 (0.3)	1.0 (3)	N/A
	Normal	98 (19.8)	27 (17.6)	71 (20.8)	N/A	N/A
	Overweight	189 (38.2)	60 (39.2)	129 (37.7)	N/A	N/A
	Obese	206 (41.6)	65 (42.5)	141 (41.2)	N/A	N/A
Physically active (yes), n (%)		228 (45.2)	78 (51.0)	150 (43.7)	2.2 (1)	N/A
Parents with diabetes (yes), n (%)		327 (65.5)	106 (69.3)	221 (64.4)	1.1 (1)	N/A
Siblings with diabetes (yes), n (%)		167 (33.2)	54 (35.3)	113 (32.9)	3.4 (1)	N/A
**Risk score, mean (SD)**		9.2 (4.7)	8.9 (4.7)	9.3 (4.7)	N/A	0.9 (494)
	<9	187 (37.7)	61 (39.9)	126 (36.7)	0.4 (1)	N/A
	>9	309 (62.3)	92 (60.1)	217 (63.3)	N/A	N/A

^a^*P*<.001.

^b^N/A: Not applicable.

The results showed that 185 individuals had a risk score≥9 but elected not to get a blood test or participate further. Most stated that they did not have time (25/185, 13.5%) or already knew that they had T2DM (21/185, 11.4%), whereas 9% (17/185) had a scheduled appointment but did not show up, 31% (57/185) gave an incorrect phone number or did not provide one, and 23% (43/185) did not answer their phone after multiple attempts.

An invitation to visit a diabetes center (Dasman Diabetes Institute) for a diagnostic blood test and enroll oneself in a prevention study based on the high risk score was accepted by 121 participants (Table 3). Of those who were invited to participate in a prevention program and get a blood test, 62.8% (76/121) were recruited through a shared SMS message through family and friends (39/76, 32.2% by word of mouth; 35/76, 28.9% via WhatsApp; and 2/76, 1.7% by other means). The majority (8/121, 66.9%) of these participants were females. Also, those between the ages of 45 and 65 (56/119, 47.1%), those with BMI scores≥30 (75/119, 63.0%), and those who were mostly Kuwaiti citizens (69/121, 57.0%) tended to pursue further testing. Most of these participants were iPhone users (95/121, 78.5%). Of the 121 individuals who consented to seek a blood test and participate in a diabetes prevention program, no differences were found between those who were initially recruited through 1 of the 5 initial text messaging campaigns (n=45) and those who were notified through family and friends (n=76).

Among the 520 respondents who completed the screening survey, differences were examined between those who agreed to be enrolled in a diabetes prevention program (n=121) and those who did not (n=399; Table 3). Those who were enrolled tended to be overweight, physically less active, and more likely to belong to the age bracket of 45-65 years compared with those who did not participate in further diabetes prevention (*P=*.034).

**Table 3:** Comparison of individuals who were eligible for enrollment in a diabetes prevention program (risk score>9) and agreed to be contacted (n=121) and those who did not enroll in the program (n=399).

Variables				Consented and enrolled (n=121), n (%)		Did not enroll (n=399), n (%)		Differences between consented and enrolled, and did not enroll		
								Chi-square (*df*)		*t* test (*df*)
Gender (female), n (%)				81 (66.9)		269 (67.0)		1.7 (2)		N/A^a^
**Age group (years), n (%)**										
		≥21-<45	62 (52.1)		240 (60.1)		6.8 (2)^b^		N/A	
		45-65	56 (47.1)		144 (35.9)		N/A		N/A	
		>65	1 (0.8)		15 (4.0)		N/A		N/A	
**BMI, mean (SD)**				32.3 (5.5)		28.9 (6.0)		N/A		5.6 (493)^c^
	Underweight, n (%)		0 (0)		2 (0.5)		40.1 (3)^c^		N/A	
	Normal		4 (3.4)		94 (25.0)		N/A		N/A	
	Overweight		40 (33.6)		149 (39.6)		N/A		N/A	
	Obese		75 (63.0)		131 (34.8)		N/A		N/A	
Physically active, n (%)				24 (19.8)		213 (53.1)		42.1 (2)^c^		N/A
Parents with diabetes, n (%)				86 (71.1)		258 (64.7)		4.1 (2)		N/A
Siblings with diabetes, n (%)				51 (42.1)		124 (30.8)		6.3 (3)		N/A
Risk score, mean (SD)				12.3 (2.4)		8.2 (4.9)		N/A		8.7 (494)^c^

^a^N/A: Not applicable.

^b^*P*=.34.

^c^*P*<.001.

## Discussion

The results of this study suggest that simple SMS messaging applied to a large population of individuals can be a feasible method of reaching a subpopulation of individuals at risk for T2DM. Past studies have demonstrated that SMSs can be used as effective health care reminders and as encouragement to change behaviors, namely for health promotion and disease prevention [25,26]. This study represents one of the first attempts to use text messaging to reach a large population of smartphone users in the Middle East for health care risk screening who might not have otherwise been identified and informed. Although further efforts are needed to refine messaging methods in order to improve acceptability, a surprise finding of this study was the role that social media played in forwarding the initial message to friends and family who might be open to prediabetes screening.

Previous studies have demonstrated that SMS messaging is a feasible, acceptable, and easy way to reach many individuals through their mobile phones and to integrate these services within the health care system [34]. This type of message campaign can be a cost-effective, convenient means to reach many individuals [35-38]. The screening experience using SMS messaging in this study was designed to reach 6% of the targeted adult population; thus, it covered a large area of Kuwait’s 6 main governorates. Despite the number of texts sent, only 0.14% (260/180,000) of the subscribers responded to the SMS messaging. It could be concluded that the messaging campaign had limited acceptability, although considering that many likely dismissed the message because they did not have concerns about diabetes, the messaging seems to have reached some who would not have otherwise enquired about their diabetes risk. This study also shows that the impact seemed to extend beyond the use of the messaging. This notion is evidenced by the number of completed surveys submitted based on word of mouth and circulated texts on social media (eg, via WhatsApp). People can forward text, audio, and video messages at no cost. For instance, unlike SMS, WhatsApp offers the use of unlimited characters and information, which could help circulate the survey more widely. Further, people can use their own words to explain their interest and experience in participating in the survey, which would be perceived to be more reliable and trustworthy [39].

This study was unable to obtain data about the number of subscribers who either did not receive the message or did not read it, or how many read the message but did not reply. Unfortunately, the telephone company used in this study could not determine this information. Quite possibly many individuals were reluctant to respond because they may have distrusted the message. Surprisingly, completed surveys continued to appear well past the time the messages were originally sent. Anecdotal information suggests that some candidates circulated the text message surveys to family members and friends, mainly via the use of other social media (predominantly WhatsApp) after establishing the legitimacy of the survey.

Several reasons might account for the relatively low response rate of the different test messaging campaigns. The selected timing of each campaign played a key role in the acceptability of and responses to the messaging. The delivery of the first 36,000 messages (Campaign 1) faced some technical difficulties with the telecommunication company, which may have accounted for many not receiving the text messages. After resolving these issues, Campaign 2 was conducted close to the time of national holidays in Kuwait (National and Liberation Days). Many Kuwait residents are known to spend this time of the year outside the country. The delivery of the messages in Campaign 3 coincided with the month of Ramadan, and many candidates responded that they would prefer to be contacted after Ramadan. Campaign 4 was initiated around the time not affected by national holidays and events, which might have helped improve its response rate compared to those of the other campaigns (113/260 total responses, 43.5%). We believe that private and public school holidays may have affected the lower response to Campaign 5. In Kuwait, private schools usually close for the Christmas holidays, and this campaign also took place around the examination time for higher education institutes. It is also important to note that replying to the screening SMS was subject to charges payable by the subscriber, which might have been an additional reason that some chose to not reply.

The majority of those who completed the survey were females, had parents or first-degree relatives with T2DM, and were overweight or obese. Thus, these responders could have had their own personal concerns about developing diabetes. Most respondents were not working or had flexible occupations, which might have given them more spare time to respond to messages on their mobile phones. We do not know if a less prestigious diabetes center (other than the Dasman Diabetes Institute) would have limited the interest and acceptability of the messages even further. Although the SMS campaigns did not meet the anticipated impact, the number of completed surveys received was doubled, as people forwarded their SMS messages to family members and friends. Receiving the message from a trusted person rather than an anonymous source might have reduced worries about the message being spam or fraudulent. This notion is corroborated by the number of individuals who were interested in completing the survey but who decided to not provide their contact numbers, who chose to enter invalid numbers, or who declined to be contacted in order to protect their identity or privacy. These individuals may have wanted to learn about their risk for diabetes but may have had privacy concerns or may have decided to pursue information from their own health care providers. It is also noteworthy that those over 65 years tended not to participate (only 17/520 or 3% of the respondents were aged 65 or more) even though they are at higher risk for diabetes compared with younger individuals [3] and would benefit from early detection [40]. This result might be attributed to older people not using text messaging as often as the younger participants.

Individuals who took the time to initiate telephone conversations expressed initial concerns about the source of the message and the link. Some were hesitant to access the link and to fill out the survey, especially since their mobile number was requested. Other factors, including the perception that the SMS messages were focused on diabetes-related complications rather than the risk of developing the disease itself, might have contributed to lower response rates. Thus, selecting the appropriate wording in a limited character text message with limited information may directly affect the comprehension of the message content, especially among those with low health literacy [41-44]. Moreover, it has been suggested that people may not be willing to initiate contact about prevention interventions when they feel healthy [45,46]. Carefully scripting the message and the public relations campaign prior to beginning the survey might likely have improved the response rates. In addition, improvements in the user interface and the message presentation designed to capture the recipient’s attention and use of a 2-way message pathway might have increased the motivation to reply and could have enhanced the response rate. A major finding of this study is that, once sent, messages can reach others by being forwarded by family members and friends through social media. This appears to be a cost-free means to improve contact with those who may be at risk for developing a chronic disease.

There are several limitations of this study that need to be discussed. First, this trial was not controlled, and we did not compare a text message campaign with other types of informational campaigns (eg, via mail or telephone). Second, Kuwait has one major phone company, which cooperated in participating in this messaging study. Researchers in other countries may not have the same accessibility to mobile phone numbers. Third, this study reached only a limited percentage of the Kuwaiti population, and only persons owning a compatible mobile phone were included. The results might have been different if other persons or areas were included. Fourth, we were unable to determine why many individuals chose to not respond to the message. The limited number of words and characters allowed in each message, timing of the messages, charges applicable when responding to the message, sending of the messages without repetitions or reminders, and general distrust of random messages may have affected the response rate. Furthermore, the abundant nature of commercial messages may have hindered the effectiveness of our SMS messaging campaigns. Finally, we used the validated CDC prediabetes screening questionnaire, which does not assess dietary patterns. Dietary habits were assessed only among those who chose to participate in the prevention program, and thus, future investigations should include an assessment of dietary habits as part of the screening.

Despite these limitations, this study demonstrates that future use of SMS health campaigns for prediabetes screening could be a feasible way to reach some at-risk individuals. Effective solutions are needed to maximize the acceptability and effectiveness of these behavioral health campaigns. Future efforts designed to help understand and improve the response rates of SMS messaging to effectively reach those individuals who are at risk for developing a chronic medical condition are needed.

## Abbreviations

CDC: Centers for Disease Control and Prevention
REDCap: Research Electronic Data Capture
T2DM: type 2 diabetes mellitus
